# Corrigendum to “Analysis of therapeutic effect of silver‐based dressings on chronic wound healing”

**DOI:** 10.1111/iwj.70049

**Published:** 2024-09-13

Liang K, Liu Y, Jiang F. Analysis of therapeutic effect of silver‐based dressings on chronic wound healing. Int Wound J. 2024;21 (8):e70006. doi:10.1111/iwj.70006

There was an error in the labelling of Figure 2. Below is the correct figure.

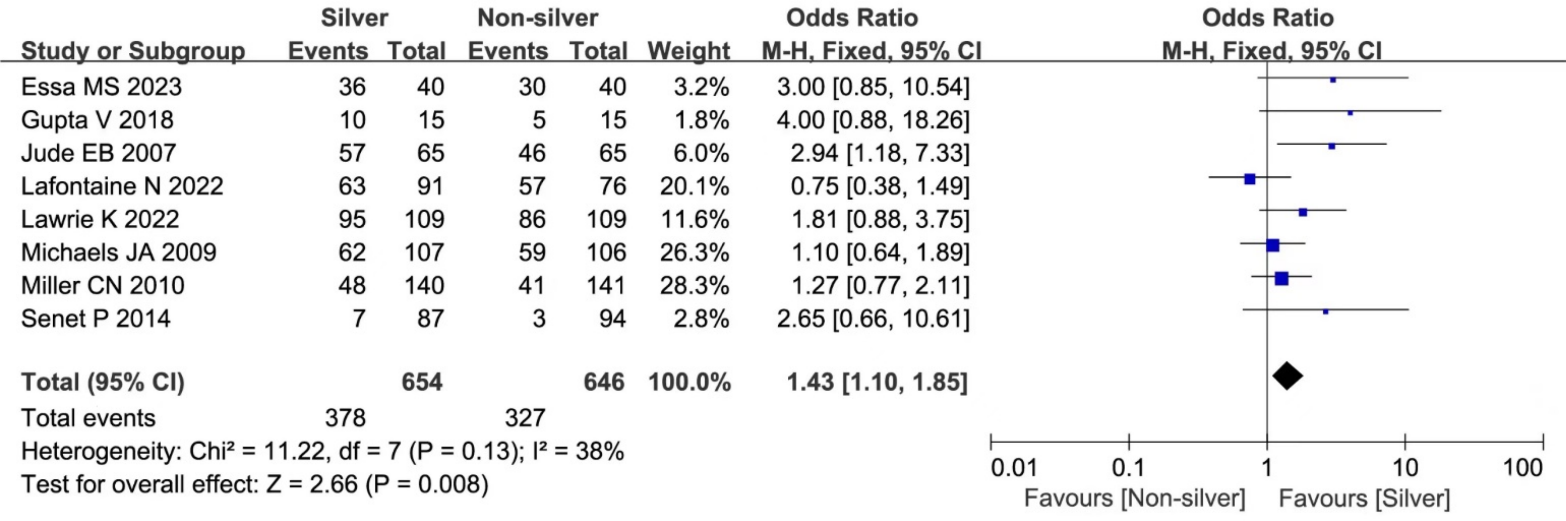

We apologize for this error.

